# Salvage nivolumab plus temozolomide associated with improved survival and clinical benefit in refractory melanoma: a retrospective cohort study

**DOI:** 10.3389/fonc.2026.1965090

**Published:** 2026-10-13

**Authors:** Preethy E. Abraham, Kun Bai, Sheau-Chiann Chen, Douglas B. Johnson

**Affiliations:** 1 Vanderbilt University School of Medicine, Nashville, TN, United States; 2 Department of Biostatistics, Vanderbilt University Medical Center, Nashville, TN, United States; 3 Department of Heme-Onc, Vanderbilt University Medical Center, Nashville, TN, United States

**Keywords:** chemotherapy, immune checkpoint inhibitor, immunotherapy, melanoma, salvage therapy, survival

## Abstract

**Introduction:**

Patients with metastatic melanoma who progress on standard of care therapy have limited treatment options. This study evaluates the efficacy of nivolumab combined with temozolomide compared to temozolomide alone in this refractory setting.

**Methods:**

We conducted a retrospective cohort study of 44 patients with metastatic melanoma who progressed on prior immunotherapy and systemic therapies. Patients were chemotherapy-naïve and treated at a single center between 2020-2025. Endpoints included objective response rate (ORR), clinical benefit rate (CBR), overall survival (OS), and progression-free survival (PFS).

**Results:**

Patients treated with nivolumab/temozolomide had improved CBR by over fourfold compared with temozolomide alone. (OR 4.276, 95% CI:1.198-15.268; p=0.025). Median OS was 11.8 months (95% CI: 6.02, NA) with nivolumab/temozolomide versus 5.49 months (95% CI: 1.91-8.97) with monotherapy. Combination therapy was also associated with a lower hazard of death (HR 0.44; 95% CI: 0.205-0.923; p=0.030) and trended toward a lower hazard of progression (HR; 0.531 CI: 0.280-1.008; p=0.053). Exploratory analysis showed a significant treatment-LDH interaction was identified for OS (p=0. 043). Within the subgroup of patients with a baseline LDH over 2 times the upper limit of normal, combination therapy was associated with a significantly reduced hazard of death (HR: 0.183; 95% CI: 0.049-0.690; p=0.012) and progression (HR: 0.205; 95% CI: 0.060-0.692; p=0.011). Nivolumab/temozolomide had numerically higher ORR (36% vs. 10.5%, p=0.053). Combination therapy demonstrated a significantly higher CBR among patients with BRAF mutations compared to monotherapy (90.0% vs. 20.0%, p=0.017).

**Conclusions:**

Nivolumab and temozolomide combination therapy may represent a viable salvage regimen for refractory melanoma.

## 1 Introduction

Immunotherapy, particularly anti-Programmed Cell Death Protein (PD-1) targeted agents, has revolutionized melanoma therapy and is the first-line treatment option for patients with advanced melanoma (1). Anti-PD-1 is often given alone or in combination with anti-Cytotoxic T-Lymphocyte-Associated Protein 4 (CTLA-4) such as ipilimumab. These immunotherapy regimens have demonstrated objective responses in 40-60% of patients, with many experiencing durable responses that can persist beyond 5–10 years (2–4). However, approximately half of the patients are refractory to these therapies, thus necessitating exploration of alternative treatment modalities (2, 5).

For patients who are refractory to immunotherapy and other available treatments, cytotoxic chemotherapy is an option but has shown limited efficacy and has not been associated with overall survival benefit (6–8). A retrospective multicenter study demonstrated that chemotherapy after checkpoint inhibitor failure yields low objective response rates (ORR), ranging from 10-19%, which are generally transient (6). Common chemotherapy regimens include dacarbazine, temozolomide, carboplatin/paclitaxel, and taxanes, but none have thus far demonstrated substantial long-term benefit.

While there is limited data at present, the combination of chemotherapy and anti-PD-1 has shown promise in enhancing response and outcomes across several cancers, as chemotherapy can modulate the tumor microenvironment and potentially improve the efficacy of immunotherapy (9–11). The synergy is driven through several mechanisms, most notably through inducing cell death and the release of damage-associated molecular patterns (DAMPs), upregulating MHC genes, and inducing expression of immune checkpoint molecules like PD-L1 (12–15). This finding has transformed treatment options for several cancers. In non-small cell lung cancer (NSCLC), large, randomized trials and meta-analyses show that anti-PD-1 with chemotherapy significantly improves overall survival (OS), progression-free survival (PFS), and response rates compared with chemotherapy alone (16–21). Similarly, chemotherapy and anti-PD-1 have improved multiple clinical endpoints in urothelial carcinoma, triple-negative breast cancer, and head and neck squamous cell carcinoma (22–25).

The utility of chemo-immunotherapy in melanoma, however, remains less well-defined, and the optimal regimen is even less well-studied. One small front-line study suggested that the addition of carboplatin and paclitaxel to pembrolizumab did not improve survival compared with pembrolizumab alone (26). The PROMIT study enrolled 38 patients who progressed on anti-PD-1 based therapy and gave two doses of dacarbazine before resuming the same anti-PD-1 regimen and noted an 18% response rate (27). Among studies of concurrent combination therapy in refractory melanoma patients, Vera Aguilera et al. examined a heterogenous, predominantly taxane/platinum-based regimen and reported improved survival and response (28). Li et al. evaluated anti-PD-1 agents combined with nab-paclitaxel in a Chinese population, and described ORR of 13.8% in refractory patients (29). A further case series by our group showed deep and durable responses in a small cohort of heavily pre-treated patients with nivolumab and temozolomide (30). However, this specific combination has not been evaluated beyond these initial observations. Temozolomide demonstrates many properties that could enhance anti-PD-1 efficacy such as depleting regulatory T cells (Tregs) and inducing T-cell tumor infiltration (31, 32). Thus, we sought to characterize the effectiveness of nivolumab/temozolomide in immunotherapy refractory metastatic melanoma patients.

## 2 Methods

### 2.1 Patients and study design

We conducted a single-center retrospective cohort study of adult patients with metastatic melanoma treated at Vanderbilt University Medical Center between January 2020 and December 2025. The study was approved by the Vanderbilt University Medical Center institutional review board (IRB #150625), which waived the requirement for informed consent due to the de-identified nature of the data. Inclusion criteria consisted of age ≥18 years, histologically confirmed cutaneous, acral or mucosal melanoma, and documented disease progression following at least one prior line of anti-PD-1-based regimen (alone or in combination with CTLA-4 and/or LAG-3 inhibition), as well as any other available systemic treatments. Patients with uveal melanoma or who had received prior chemotherapy were excluded (33, 34).

Eligible patients were assessed based on whether they received nivolumab and temozolomide or temozolomide alone between January 1, 2020, and December 31, 2025. The choice of combination therapy versus chemotherapy alone was based on physician preference and evolved over the study period. Temozolomide alone predominated earlier, and combination therapy was adopted progressively after early responses were observed, except in patients with previous severe immune-related adverse events (irAEs). All patients received temozolomide dosed at 200mg/m^2^ days 1–5 of a 28-day cycle. Nivolumab was given 240 mg IV every 2 weeks or 480 mg every 4 weeks. Retrospective clinical data were collected, including patient demographics, comorbidities, and prior treatment details. Tumor characteristics were documented, specifically mutation status, date of initial metastatic disease and date of progression prior to nivolumab/temozolomide initiation. AJCC stage, M category and sites of metastatic disease at the time of study treatment initiation were collected. Adverse events, including irAEs, hematologic toxicities, and other treatment-emergent adverse events, were obtained by chart review and graded according to the Common Terminology Criteria for Adverse Events (CTCAE) version 5.0. Treatment cycles, dose reductions, delays, discontinuations, and hospitalizations were also recorded.

The primary endpoint was objective response rate (ORR), assessed radiographically according to Response Evaluation Criteria in Solid Tumors (RECIST) version 1.1. Objective responses (complete or partial response) were considered confirmed if maintained on a subsequent imaging assessment performed no less than 4 weeks after the criteria for response were first met. In this cohort, confirmatory imaging was obtained at the next scheduled evaluation (approximately 8 weeks). All radiographic response assessments were performed by the treated physician, who was not blinded to treatment assignment. Radiographic evaluation was performed per institutional standard of care, generally every 8 weeks from treatment start. Secondary endpoints included clinical benefit rate (CBR), PFS and OS. PFS was defined as the time from treatment initiation to progression (per RECIST 1.1) or death from any cause. Patients without an event at the data cutoff, or who were lost to follow-up, were censored at the date of last contact. OS was defined as the time from treatment initiation to death from any cause.

### 2.2 Statistical analysis

Descriptive statistics were summarized as mean, median, standard deviation and interquartile range (IQR) for continuous variables, and as frequency for categorical variables for the overall cohort and by treatment groups. Demographic and clinical factors were compared between treatment groups. For the two group comparisons, Kruskal-Wallis test was used for continuous variables. Pearson’s Chi-squared test/Fisher’s exact test was applied to evaluate associations between categorical variables. Variables compared include treatment response, CBR, prior treatment exposure, disease stage, BRAF mutation status, baseline LDH levels (in both continuous and categorical relative to the upper limit of normal [ULN; 220 U/L]: normal 1-2x ULN, and >2 ULN), absolute lymphocyte count (ALC) and neutrophil to lymphocyte ratio (NLR) at baseline and early treatment timepoints. Patients with missing baseline data for specific variables (e.g., LDH) were excluded from those specific subgroup or interaction analyses.

Univariate logistic regression models were utilized to evaluate the effect of treatment assignment on ORR and CBR. Effect sizes were estimated using odds ratios (OR) and 95% confidence intervals (CI). OS and PFS were estimated using the Kaplan-Meier method and compared using the log-rank test in survival plots. Median OS and median PFS, along with their 95% CIs, were reported. Univariable Cox proportional hazards regression models were utilized to estimate hazard ratios (HR) and 95% CI. P value for HR was based on the Wald test, which assesses whether the Cox regression coefficient differs significantly from zero (i.e., whether HR = 1). To address potential small-sample bias and sparse-event issues, penalized maximum likelihood estimation with a Jeffreys-prior penalty (Firth’s method) was used for logistic regression (35) and Cox proportional hazards regression (36). This approach reduces estimation bias and helps obtain stable, finite parameter estimates when the number of events is low, or data separation occurs.

For exploratory analyses, multivariable Cox regression models with Firth’s penalized likelihood were conducted to assess potential two-way interaction effects between treatment group and each key clinical covariate (prior treatment exposure, disease stage, and baseline LDH levels). The primary interaction analyses modeled LDH as a continuous log-transformed variable to account for its right-skewed distribution and to avoid information loss associated with categorization. Clinically relevant LDH categories that standardize LDH relative to the laboratory ULN, were additionally evaluated to enhance interpretability and facilitate comparison of treatment effects across patient groups commonly used in clinical practice. Interaction effects were evaluated using the likelihood ratio test comparing models with and without the interaction term. Treatment effects were evaluated across the observed LDH levels. The estimated ORs or HRs with 95% CI were reported over LDH levels. No formal adjustment for multiple comparisons was applied.

Prior to multivariable modeling, variable clustering using Spearman’s rank correlation coefficient and redundancy analyses with an *R*^2^ threshold of 0.8 were performed to assess potential multicollinearity among clinical covariates. No major clusters indicating strong collinearity were observed, and no redundancy was identified. All statistical analyses were conducted using R v4.5.1.

## 3 Results

### 3.1 Patient characteristics

A total of 44 patients [16 female (36.4%); mean (SD) age, 60.8 (16.0) years] were included (**Table 1**). Of these, 19 patients were treated with temozolomide alone and 25 patients received nivolumab and temozolomide. Baseline demographic and clinical characteristics were similar between the two cohorts. The population had a median of 2 prior lines of systemic therapy (IQR 2.0-4.0 for nivolumab and temozolomide vs. 2.0-3.0 for temozolomide alone, p=0.705). Most patients had advanced metastatic burden at the time of treatment initiation, with M1c/M1d stage disease present in 78.9% of the monotherapy group and 84% of the combination group (p=0.667). Among patients with known mutational status (n=42), BRAF mutations were present in 10 (41.7%) in the combination group and 5 (27.8%) in the monotherapy group (p=0.517); all had previously progressed on BRAF/MEK inhibitors. Baseline LDH levels were similarly elevated in both groups (mean 501.00 IU/L vs. 475.75 IU/L; p=0.990). Almost one-third of patients had a baseline LDH value >2x ULN.

**Table 1:** Baseline clinical and demographic characteristics of the study cohort.

	Temozolomide (N = 19)	Nivolumab/temozolomide (N = 25)	Total (N = 44)	p value
Age at treatment start				0.627
Mean (SD)	59.37 (16.18)	61.96 (16.17)	60.84 (16.03)	
Median (IQR)	62.0 (42.5, 75.0)	62.0 (55.0, 77.0)	62.0 (48.5, 76.2)	
Gender				0.490
F	8 (42.1%)	8 (32.0%)	16 (36.4%)	
M	11 (57.9%)	17 (68.0%)	28 (63.6%)	
Number of prior treatments				0.705
Mean (SD)	2.74 (1.9)	2.80 (1.5)	2.80 (1.6)	
Median (IQR)	2.00 (2.00, 3.00)	2.00 (2.00, 4.00)	2.00 (2.00, 3.00)	
Stage at Treatment Start (M1a-d)				0.667
M1a+M1b	4 (21.1%)	4 (16.0%)	8 (18.2%)	
M1c+M1d	15 (78.9%)	21 (84.0%)	36 (81.8%)	
BRAF Status*				0.517
Mutant	5 (27.8%)	10 (41.7%)	15 (35.7%)	
Wild type	13 (72.2%)	14 (58.3%)	27 (64.3%)	
Baseline LDH **				0.990
Mean (SD)	501.0 (627.02)	475.75 (434.96)	486.91 (521.78)	
Median (IQR)	337.00 (225.50, 496.00)	326.00 (228.50, 563.00)	334.0 (226.00, 515.00)	
LDH Group **				0.668
Normal	5 (26.3%)	4 (16.7%)	9 (20.9%)	
1-2x ULN	8 (42.1%)	13 (54.2%)	21 (48.8%)	
2+ ULN	6 (31.6%)	7 (29.2%)	13 (30.2%)	

Data are presented as n (%) or median (IQR) as indicated. IQR, interquartile range. LDH, lactate dehydrogenase. ULN, upper limit of normal.

*BRAF Status unavailable for one patient in both the Nivolumab/Temozolomide group (N = 24) and in the Temozolomide group (N = 18).

**LDH data unavailable for one patient in the Nivolumab/Temozolomide group (N = 24).

Objective responses occurred in 2 of 19 patients (10.5%) treated with temozolomide alone and 9 of 25 patients (36%) who received nivolumab and temozolomide (**Table 2**). Of these, 1 patient in the monotherapy group had a confirmed objective response on subsequent radiographic evaluation, compared with 5 patients in the combination group. Notably, the CBR was 31.6% (6/19) for temozolomide and 68% (17/25) for the combination (p=0.017). Individual patient responses, documented as change in aggregate diameter of measurable tumors, for patients treated with combination therapy are shown in **Figure 1**.

**Table 2:** Objective response and clinical benefit rates by treatment cohort.

	Temozolomide (N = 19)	Nivolumab/temozolomide (N = 25)	Total (N = 44)	p value
Response (CR+PR)				0.053
No	17 (89.5%)	16 (64.0%)	33 (75%)	
Yes	2 (10.5%)	9 (36.0%)	11 (25%)	
Clinical Benefit Rate (CBR)				0.017
No	13 (68.4%)	8 (32.0%)	21 (47.7%)	
Yes	6 (31.6%)	17 (68.0%)	23 (52.5%)	

Data are presented as n (%). CR, complete response. PR, partial response.

**Figure 1:**
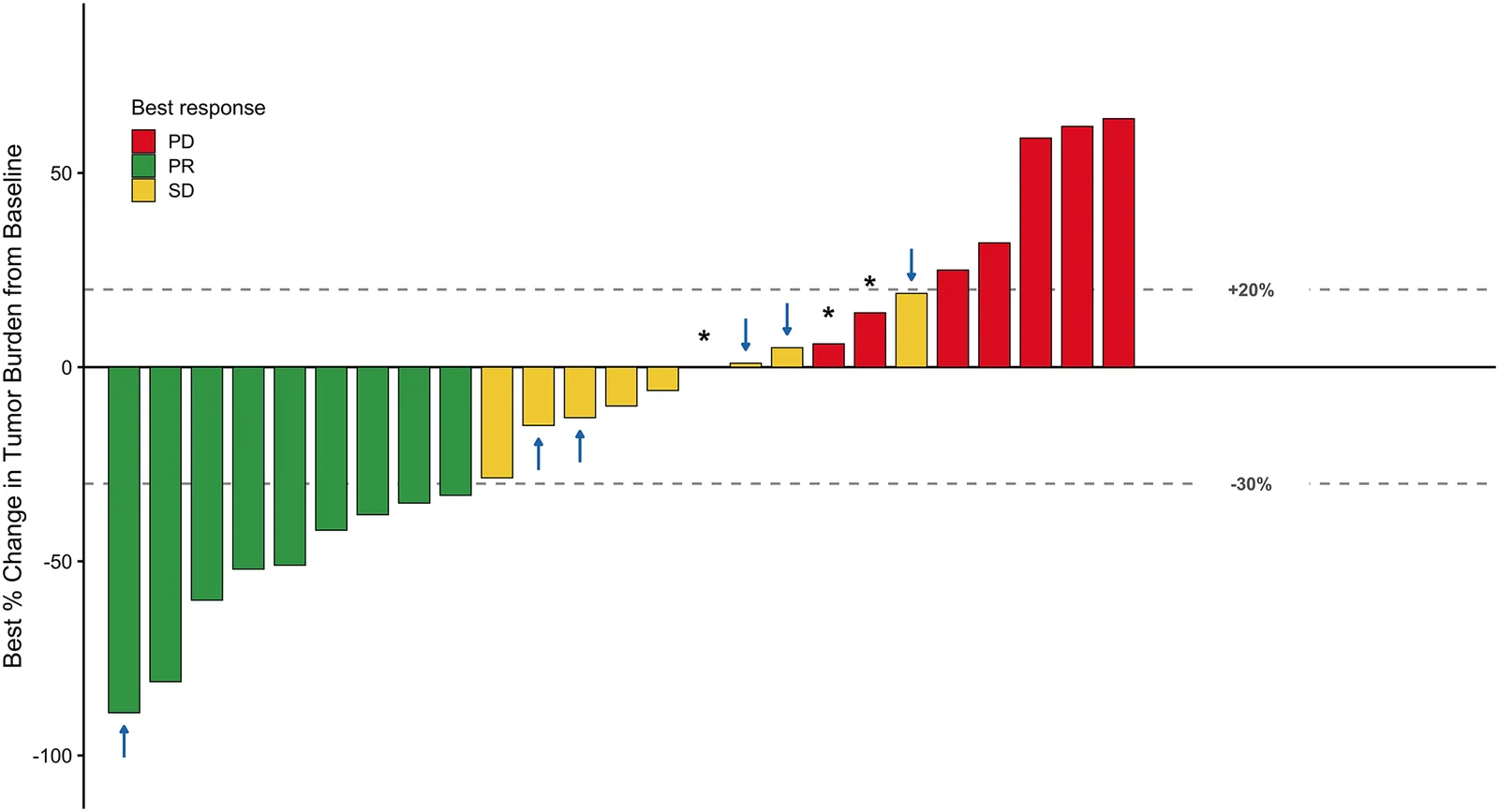
Best objective response to nivolumab/temozolomide. Waterfall plot illustrating the maximum percentage change from baseline for each evaluable patient, assessed per RECIST 1.1 criteria. Each bar represents one patient. Bars are color-coded by best overall response: green for partial response (PR), yellow for stable disease (SD), and red for progressive disease (PD). Asterisks (*) denote patients who experienced clinical deterioration or development of new lesions. Blue arrows indicate patients with mucosal melanoma. No patient experienced complete response (CR). PD, progressive disease; PR, partial response; SD, stable disease.

### 3.2 Univariate analysis

#### 3.2.1 Response and clinical benefit rate

In univariate logistic regression with Firth’s penalized likelihood, combination nivolumab and temozolomide therapy was associated with an odds ratio of 4.030 for response (95% CI: 0.826-19.659; p=0.085). For CBR, the combination regimen was associated with an odds ratio of 4.276 (95% CI: 1.198-15.268; p=0.025).

In subgroup analysis, BRAF mutation status was associated with response in the combination group. Within the nivolumab and temozolomide group, patients with BRAF mutations had numerically higher ORR (50.0% vs. 21.4%, p=0.204) and CBR (90.0% vs. 50%, p=0.079) compared to those with wild-type status. In patients with BRAF mutations, ORR was also numerically higher for nivolumab and temozolomide compared to temozolomide monotherapy (50.0% vs. 0%, p=0.101). The CBR was significantly higher (90.0% vs. 20.0%, p=0.017). In the BRAF wild-type population, no treatment-related differences were observed for either ORR (21.4% vs. 15.4%, p=1.00) or CBR (50% vs. 38.5%, p=0.704). Notably, we also observed several patients with clinically meaningful mixed responses (e.g., **Supplementary Figure S1**) that allowed for decreased opiate requirements or other clinical benefits.

For patients in the nivolumab and temozolomide group, the NLR at baseline was significantly lower in responders versus non-responders (median 3.11 vs 5.07; p=0.011). Baseline ALC, Week 2 ALC, and week 4 NLR, week 2 to baseline ALC fold-change, and the absolute change in NLR at weeks 2,4 and 8 were not associated with response (p > 0.05). In the temozolomide group, similar analysis could not be completed due to an insufficient number of responders with available laboratory data at these timepoints.

#### 3.2.2 Overall survival

The median OS for all patients was 8.98 months (CI: 5.29-11.8), with 28 deaths among 44 patients before the last follow-up. Survival was significantly longer in the combination group, with a median OS of 11.80 months for combination therapy (CI: 6.02-NA) and 5.49 months (CI: 1.91-8.97) for temozolomide alone (p=0.030; Firth’s Cox penalized likelihood ratio test) (**Figure 2A**). Univariate Firth’s Cox proportional hazards regression demonstrated that combination therapy with nivolumab and temozolomide resulted in a significant reduction in the hazard of death compared to temozolomide monotherapy (HR = 0.44; 95% CI: 0.205-0.923; p=0.030). We also evaluated clinical covariates individually; baseline log LDH levels (p=0.134), disease stage (p=0.282) and prior systemic treatment (p=0.176) were not significantly associated with survival.

**Figure 2:**
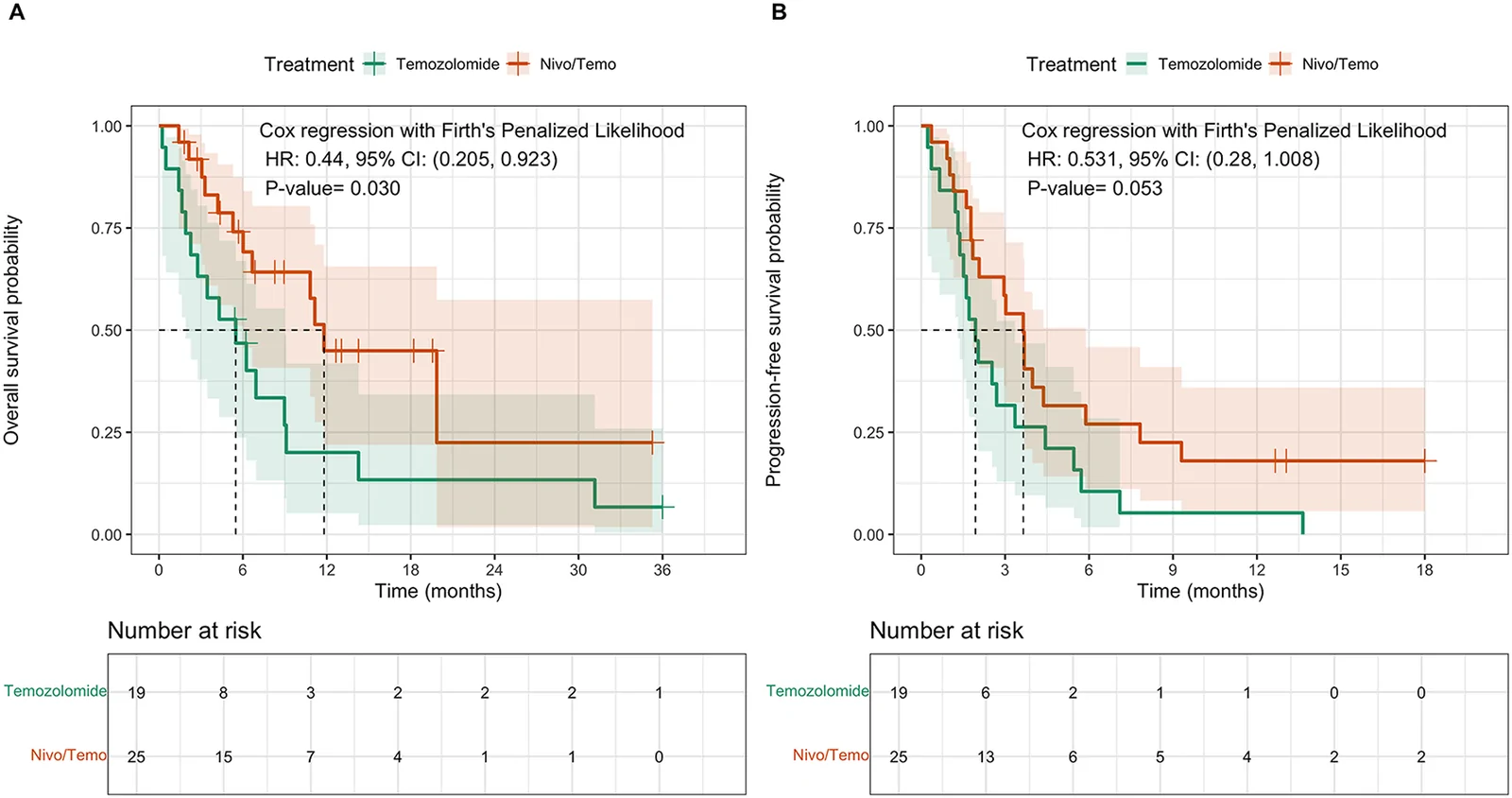
Survival outcomes by treatment cohort. **(A)** Overall survival (OS) and **(B)** progression-free survival (PFS) by treatment cohort. Kaplan-Meier estimates of OS and PFS for patients receiving nivolumab/temozolomide (Nivo/Temo) (n=25) versus temozolomide monotherapy (n=19). Shaded regions represent 95% confidence intervals, dashed lines indicate median survival, and tick marks denote censored observations. Hazard ratio (HR), 95% confidence interval (CI), and P-value were reported using Cox regression with Firth’s penalized likelihood. The x-axis was presented until 3 years for OS and 1.5 years for PFS because few patients remained at risk beyond these time points; no additional OS or PFS events occurred thereafter.

#### 3.2.3 Progression free survival

The median PFS for all patients was 2.70 months (CI:1.78-3.68), with 38/44 patients experiencing disease progression. The median PFS was 3.65 months for combination therapy (CI: 1.84-5.88) compared to 1.94 months for monotherapy (CI: 1.31-3.35) (p=0.053, Firth’s Cox Penalized likelihood ratio test) (**Figure 2B**). The HR for combination nivolumab and temozolomide was 0.531 (95% CI: 0.280-1.008), which approached but did not reach statistical significance (p=0.053). Evaluation of clinical covariates revealed that disease stage was significantly associated with PFS (p=0.040). Prior systemic therapy (p=0.709) and baseline log LDH levels (p=0.815) were not statistically significant.

### 3.3 Multivariable analysis

#### 3.3.1 Overall survival

The association between OS and each potential treatment-covariate interaction was explored. A significant interaction between treatment and baseline LDH was identified (likelihood ratio test; p=0.04245). Across the continuous range of LDH values, the hazard ratio for nivolumab plus temozolomide versus temozolomide alone decreased with increasing LDH levels (**Supplementary Figure S2**), indicating greater treatment benefit among patients with elevated LDH. This finding was supported by categorical analyses, which demonstrated progressively lower hazard ratios across increasing LDH strata: 0.750 (95% CI: 0.099-5.693, p=0.780) for LDH ≤ULN, 0.472 (95% CI: 0.158-1.411, p=0.179) for LDH 1-2× ULN, and 0.183 (95% CI: 0.049-0.690, p=0.012) for LDH >2× ULN.

#### 3.3.2 Progression free survival

Exploratory analyses suggested a potential interaction between treatment and baseline LDH for PFS (likelihood ratio test, p=0.099). Across the observed range of baseline LDH values, the hazard ratio for nivolumab plus temozolomide versus temozolomide alone decreased as LDH increased, suggesting a greater treatment benefit among patients with elevated LDH (**Supplementary Figure S3**). Consistent with this pattern, treatment effects estimated within clinically relevant LDH categories demonstrated increasingly favorable hazard ratios with increasing LDH levels. The HR for combination therapy was 0.790 (95% CI: 0.191-3.268, p=0.745) among patients with LDH within normal limits, 0.732 (95% CI: 0.288-1.863, p=0.513) among those with LDH 1-2× ULN, and 0.205 (95% CI: 0.060-0.692, p=0.011) among those with LDH >2× ULN. Notably, a statistically significant improvement in PFS with combination therapy was observed only in patients with LDH >2× ULN.

### 3.4 Safety data

In the combination group, patients received a median of 4 cycles of concurrent nivolumab and temozolomide (range, 1-15). Hematologic toxicities were primarily attributed to temozolomide. This consisted of grade 3–4 leukopenia (n=4, 16%) and grade 4 thrombocytopenia (n=3, 12%). Reported irAEs were grade 4 pneumonitis (n=1, 4%), grade 3 colitis (n=1, 4%), grade 3 dermatitis (n=1, 4%), and grades 1–3 pruritus (n=3, 12%). Five patients (20%) in the combination group experienced temozolomide dose reductions and two patients (8%) experienced treatment delays (one due to toxicity and one due to patient preference). Five patients (20%) were hospitalized secondary to therapy. Two patients (8%) remain on combination therapy at the data cutoff date, six (24%) patients continued on nivolumab alone, and the remaining 17 discontinued both—13 patients (52%) due to progression, three (12%) due to toxicity, and one (4%) due to death.

In the temozolomide alone group, patients received a median of 2 cycles of temozolomide (range, 1-11). Hematologic toxicities included grade 4 thrombocytopenia (n=2, 10.5%), grade 3 leukopenia (n=2, 10.5%), and grade 3 anemia (n=1, 5.3%). Non-hematologic emergent adverse events included grade 2 pneumonitis (n=1, 5.3%). Two patients (10.5%) experienced dose reductions, and four patients (21.1%) experienced treatment delays. No patients were hospitalized secondary to temozolomide therapy. One patient (5.3%) was initiated on temozolomide while hospitalized. All patients eventually discontinued treatment due to progression (n=18, 94.7%), or death (n=1, 5.3%).

## 4 Discussion

In this study, the combination of nivolumab and temozolomide was associated with improved CBR, longer OS, and a trend toward improved ORR and PFS compared to temozolomide alone in patients with refractory melanoma. Notably, this study provides the first retrospective evidence that adding nivolumab to temozolomide offers a survival advantage even in this setting. We hypothesize that this advantage may be driven by durable disease control rather than tumor regression alone, reflected in the significant CBR, but not ORR. However, the non-significant ORR (and PFS) may reflect the limited power of our modest sample size rather than a true absence of effect.

Our findings align with meta-analytical evidence from over 16,000 patients demonstrating that novel immunotherapy or combination targeted agents significantly improve survival in poor prognostic subgroups, such as those with elevated baseline LDH (37). Specifically, we examined an exploratory interaction between treatment efficacy and elevated LDH, where the combination of nivolumab and temozolomide showed a greater apparent survival benefit for patients with aggressive, high-burden disease who typically derive little benefit from chemotherapy alone. By combining immune activation with cytotoxic therapy, this approach may mitigate the rapid disease progression typically associated with elevated LDH.

Beyond the survival interactions identified, we also investigated predictive markers. Our results suggest that BRAF mutation status may be associated with response to the combination regimen. Patients with BRAF mutations treated with the combination experienced significantly higher ORR and CBR compared to patients with wild-type BRAF. While this finding may be driven by the limited sample size and multiple comparisons, it warrants further exploration. We also assessed whether neutrophil or lymphocyte counts might be associated with response; notably, a lower NLR at baseline was significantly associated with response among patients receiving combination therapy, although other associations at subsequent weeks were not present. This finding is consistent with prior literature demonstrating that a lower NLR is associated with improved outcomes in patients treated with immune checkpoint inhibitors (38, 39).

The potential synergy between cytotoxic agents and immune checkpoint inhibitors in the refractory setting may be explained by the interplay between tumor cell death and immune activation. Preclinical data in melanoma suggests that enhancing ferroptosis can remodel the tumor microenvironment to facilitate CD8+ T-cell infiltration, providing a plausible mechanistic rationale for combining cytotoxic stress with PD-1 blockade (40). Complementary preclinical data reported that a PD-L1-SARS peptide vaccine increased intratumoral CD8+ T cells and that pairing this vaccine with anti-PD-L1 improved antitumor efficacy and survival in murine models (41). Together, these data support a broader principle that pairing an additional agent with PD-(L)-1 blockade can augment antitumor immunity.

While immune priming and activation can help overcome resistance, there is heterogeneity among resistance mechanisms in melanoma. This can include mechanisms such as high expression of peroxidasin found in invasive melanoma cells (42), or immune evasion mediated by cytokines such as macrophage migration inhibitory factor (43). The baseline immune milieu of the tumor microenvironment may also impact response, with a predominance of monocyte-derived macrophages and cytotoxic T cells associated with anti-PD-1 response whereas a predominance of suppressive macrophages was linked with non-response (44). A translational study investigating predictive biomarkers in melanoma identified a four-gene signature (CD24, NFIL3, FN1, KLRK1) whose upregulation significantly correlated with immunotherapy resistance and decreased survival (45). These mechanistic and biomarker data provide biological context for the variable responses seen in refractory disease. Further studies should examine whether these features identify which refractory patients are most likely to benefit from nivolumab and temozolomide combination therapy.

The clinical response observed in this subset is particularly notable when compared to the broader landscape of salvage therapies for PD-1 inhibitor refractory melanoma. Several therapeutic strategies have been investigated to overcome resistance to single-agent PD-1 blockade. Notably, most of these regimens include continuation or rechallenge of PD-1 inhibitors combined with additional agents, as we did in our cohort. Most notably, the SWOG S1616 trial and retrospective analyses have validated the efficacy of combining ipilimumab with PD-1 inhibition in the refractory setting (46, 47). Nivolumab and relatlimab demonstrated approximately 12% response rates in prospective trials in a heavily pre-treated population (48), though retrospective data suggests efficacy could be better in a real-world population (49). The LEAP-004 trial showed some activity for the combination of pembrolizumab and lenvatinib in a refractory population, with ORR of 21.4%, and a median duration of response of about 8.3 months (50). Finally, in the domain of cellular therapy, lifileucel has demonstrated durable responses (ORR 31.4%) in immunotherapy refractory patients, establishing it as a potential salvage option in a select group of patients with accessible tumors for TIL harvest and very robust performance status (51). With the exception of lifileucel, all of these regimens involve continued anti-PD-1 therapy despite prior progression; continuation of therapy appears to augment response to the other agent, similar to our study. Also of note, nivolumab and temozolomide appears to have comparable or even favorable efficacy compared with these salvage regimens, particularly in patients who have progressed on ipilimumab and nivolumab and for patients not eligible for lifileucel.

These findings may inform decision-making for refractory patients with few remaining options. The regimen is most relevant for patients who have progressed on prior PD-1 blockade (including ipilimumab and nivolumab) yet remain candidates for continued immunotherapy. Patients with elevated LDH may derive particular benefit. Additionally, temozolomide provides a pragmatic, oral and widely accessible cytotoxic backbone onto which PD-1 blockade can be maintained. Adverse events with the combination were consistent with the known profiles of each agent, with no unexpected or apparently additive toxicity. Hematologic events were attributable to temozolomide and were observed in the temozolomide alone group as well. Only 3 of the 25 patients (12%) discontinued the combination therapy for toxicity, supporting its tolerability in this heavily pre-treated population. This regimen may be most useful when clinical-trial enrollment is not feasible, and other salvage strategies (such as ipilimumab-based rechallenge or TIL therapy) are unavailable, contraindicated or already exhausted. It may provide an alternative for patients who might otherwise default to supportive care.

Our study has several limitations, primarily its retrospective design, small sample size, and single-institution nature. The modest sample size likely limited statistical power and increased the potential for instability in multivariate and interaction models. The single-center design may limit generalizability due to potential geographic and institutional biases in patient population and clinical practice. Additionally, the heterogeneity of prior treatments and non-randomized treatment allocation introduce potential confounding by indication. Because combination-treated patients were generally treated in a later period and were able to tolerate continued immunotherapy, the observed survival differences may be partly influenced by these selection factors. Accordingly, these findings should be regarded as hypothesis-generating. However, the baseline characteristics were similar between both groups, and the inclusion of a high-risk population with heavy disease burden strengthens the external validity of our findings. Unlike clinical trials that often exclude patients with poor performance status or extensive metastases, this real-world dataset reflects the clinically complex population encountered in routine practice and included many patients who would not/did not qualify for clinical trials.

These findings generate a compelling hypothesis that maintaining PD-1 blockade in addition to temozolomide provides a survival benefit over chemotherapy alone, particularly in patients with high-risk features who have exhausted standard of care options.

## 5 Conclusion

Combination nivolumab and temozolomide represents a feasible salvage regimen for patients who have progressed on standard immunotherapy and have exhausted alternative treatment options. By extending survival and eliciting clinical benefit in a population with aggressive clinical features, this regimen serves as an important addition to the refractory treatment landscape. Future work should further investigate whether this combination regimen is particularly beneficial in patients with elevated LDH or BRAF mutations. While prospective validation is warranted, this approach provides a viable option for real-world patients who have few remaining lines of therapy.

## Data Availability

The raw data supporting the conclusions of this article will be made available by the authors, without undue reservation.
