# Supplementary material for: Salvage nivolumab plus temozolomide associated with improved survival and clinical benefit in refractory melanoma: a retrospective cohort study
Source: Front Oncol. 2026 Oct 13;16:1965090. doi: 10.3389/fonc.2026.1965090 (PMC13709978; doi:10.3389/fonc.2026.1965090)
Supplement: Supplementary file 1 [file DataSheet1.docx]

Supplementary Material

**Supplementary Figure S1:** Computed tomography (CT) Imaging of mixed response to Nivolumab/ Temozolomide in a patient with refractory metastatic melanoma.

(A) Baseline and (B) 3 month follow up imaging of responding right hepatic lobe conglomerate, showing a marked decrease in size.

(C) Baseline and (D) 3 month follow up imaging of new left stomach wall lesion.

**Supplementary Figure S2:** Overall survival treatment effect across baseline LDH. Estimated hazard ratios (HRs) for overall survival comparing nivolumab/temozolomide (Nivo/Temo) with temozolomide alone across the observed range of baseline lactate dehydrogenase (LDH) levels. HR points (solid point) with 95% confidence interval (vertical lines) were estimated from the Cox regression model with Firth’s penalized likelihood that included a treatment-by-log (LDH) interaction. The model was fitted on the natural log scale of LDH; the x-axis shows back-transformed LDH values (units/L). The horizontal dashed line denotes an HR of 1.0, indicating no treatment effect.

**Supplementary Figure S3:** Progression free survival treatment effect across baseline LDH. Estimated hazard ratios (HRs) for progression-free survival comparing nivolumab/temozolomide (Nivo/Temo) with temozolomide alone across the observed range of baseline lactate dehydrogenase (LDH). HRs (solid circles) and 95% confidence intervals (vertical lines) were estimated from a Cox regression model with Firth’s penalized likelihood that included a treatment-by-log (LDH) interaction. The model was fitted on the natural log scale of LDH; the x-axis shows back-transformed LDH values (U/L). The horizontal dashed line denotes an HR of 1.0, indicating no treatment effect
